# A systematic comparison and evaluation of high density exon arrays and RNA-seq technology used to unravel the peripheral blood transcriptome of sickle cell disease

**DOI:** 10.1186/1755-8794-5-28

**Published:** 2012-06-29

**Authors:** Nalini Raghavachari, Jennifer Barb, Yanqin Yang, Poching Liu, Kimberly Woodhouse, Daniel Levy, Christopher J O‘Donnell, Peter J Munson, Gregory J Kato

**Affiliations:** 1 Genomics Core Facility, Genetics and Development Biology, NHLBI, The National Institutes of Health, 10 Center Drive, Bldg 10, 8C 103B, Bethesda, 20892, USA; 2 Mathematical and Statistical computing Laboratory, Center for Information Technology, National Institutes of Health, Bethesda, MD, USA; 3 Hematology Branch, National Institutes of Health, Bethesda, MD, USA; 4 The National Heart, Lung, and Blood Institute’s Framingham Heart Study, Framingham, MA, USA; 5 The Center for Population Studies and the Division of Intramural Research, National Heart, Lung, and Blood Institute, Bethesda, MD, USA; 6 The Center for Cardiovascular Genomics and the Division of Intramural Research, National Heart, Lung, and Blood Institute, Bethesda, MD, USA

**Keywords:** Sickle cell disease, RNA-Seq, Exon arrays, Transcriptome, Clinical genomics

## Abstract

**Background:**

Transcriptomic studies in clinical research are essential tools for deciphering the functional elements of the genome and unraveling underlying disease mechanisms. Various technologies have been developed to deduce and quantify the transcriptome including hybridization and sequencing-based approaches. Recently, high density exon microarrays have been successfully employed for detecting differentially expressed genes and alternative splicing events for biomarker discovery and disease diagnostics. The field of transcriptomics is currently being revolutionized by high throughput DNA sequencing methodologies to map, characterize, and quantify the transcriptome.

**Methods:**

In an effort to understand the merits and limitations of each of these tools, we undertook a study of the transcriptome in sickle cell disease, a monogenic disease comparing the Affymetrix Human Exon 1.0 ST microarray (Exon array) and Illumina’s deep sequencing technology (RNA-seq) on whole blood clinical specimens.

**Results:**

Analysis indicated a strong concordance (R = 0.64) between Exon array and RNA-seq data at both gene level and exon level transcript expression. The magnitude of differential expression was found to be generally higher in RNA-seq than in the Exon microarrays. We also demonstrate for the first time the ability of RNA-seq technology to discover novel transcript variants and differential expression in previously unannotated genomic regions in sickle cell disease. In addition to detecting expression level changes, RNA-seq technology was also able to identify sequence variation in the expressed transcripts**.**

**Conclusions:**

Our findings suggest that microarrays remain useful and accurate for transcriptomic analysis of clinical samples with low input requirements, while RNA-seq technology complements and extends microarray measurements for novel discoveries.

## Background

With the completion of the human genome project, the monitoring of changes in the entire human transcriptome is an increasingly attractive method for dissecting the molecular basis of disease processes [1,2]. In this regard, the ability to utilize a patient’s transcriptome to detect the onset of disease, monitor its progression, and even to suggest treatment modalities with the highest probability of success would greatly enhance the quality of medical care and treatment [3-6]. Peripheral whole blood is a nucleic acid-rich and inflammatory cell-rich information reservoir. Analytical methodologies to detect transcriptomic changes in these cells may reveal novel biomarkers for disease diagnosis and treatment.

Until recently, high throughput microarray technologies have been the method of choice in clinical studies with limited amounts of RNA from blood samples, biopsy specimens, or enriched cell populations, to obtain gene expression profiles. The high density Affymetrix Human Exon 1.0 ST array (Exon array) with 1.2 million probesets targeting every known and predicted exon in the entire genome has been successfully employed in clinical investigations for obtaining gene expression profiles and associated alternative splicing events in disease processes [7-9]. Despite these successes, inherent limitations in the dynamic range of arrays and the lack of complete coverage for detecting alternative splicing events have constrained the application of this technology.

With the advent of next-generation sequencing technologies, RNA-seq has emerged as a powerful tool for transcriptome analysis [10-12]. By mapping millions of RNA-seq reads to individual transcripts, estimation of expression levels of individual exons and whole transcripts can be performed. It is likely that the microarray-based (analog) gene-expression profiling technology will be replaced by digital sequencing based gene-expression profiling (RNA-seq) [8,13,14]. The purported advantages of RNA-seq include generation of expression data for individual annotated genes with nearly unlimited dynamic range; ability to comprehensively detect novel transcripts and mRNA variants resulting from alternative promoter usages, splicing, polyadenylation and sequence variation; and lowered background. However, the technology also brings with it new issues such as the requirement for large amounts of starting material, cumbersome library preparation; novel systematic biases during sample preparation and sequencing that must be accounted for when analyzing the data. Additionally, data processing of multireads and splice junctions have been problematic when mapping the sequences back to a genome.

Considering the merits and limitations of each of the technologies, we undertook the current study using complex whole blood specimens from patients with sickle cell disease (SCD) and matched healthy controls to address the following major questions:

(1). How do the technologies compare with regard to sensitivity, specificity, and variability of gene expression data? (2). Do the differentially expressed transcripts and alternatively spliced exons in SCD correlate well between RNA-seq and Exon arrays? (3). Does the abundant expression of globin transcripts in SCD interfere with RNA-seq analysis? (4). Are we able to discover novel differentially expressed transcripts in SCD using RNA-seq? (5). Can we detect sequence variants in the expressed transcripts?

Although previous comparative studies [15,16] have reported the advantages of RNA-Seq and microarrays, to our knowledge, our study is the first to use a human monogenic disease model to compare RNA-Seq and microarrays. We report here our observations on the SCD transcriptome from RNA-seq and Exon arrays with the belief that our findings will be useful to clinical investigators in choosing the appropriate genomic technique for understanding molecular mechanism of diseases for diagnosis and the development of novel therapeutics.

## Methods

### Subjects

The study was approved by the National Institute of Diabetes and Digestive and Kidney Diseases institutional review board (NIH protocol 03-CC-0015) and written informed consents were obtained from study participants. Patients selected for this study included patients (n = 6) with sickle cell disease of mean age 41.6 ± 10.1 and the control group (n = 4) of self-identified African American subjects of mean age 42.2 ± 8.9, without sickle cell disease. The biosamples were collected in steady state condition and none of the individuals was receiving anti-platelet medication.

### Isolation of RNA from whole blood specimens

Blood specimens (2.5 ml) collected in PAXgene™ tubes from each subject were incubated at room temperature for 4 h for RNA stabilization and then stored at − 80 °C. RNA was extracted from whole blood using the PAXgene™ Blood RNA System Kit following the manufacturer's guidelines. Briefly, samples were removed from −80 °C and incubated at room temperature for 2 hours to ensure complete lysis. Following lysis, the tubes were centrifuged for 10 min at 5,000 × g the supernatant was discarded and 500 μL of RNase-free water added to the pellet. The tube was vortexed thoroughly to re-suspend the pellet, centrifuged for 10 min at 5000 × g and the entire supernatant was discarded. The pellet was re-suspended in 360 μL of buffer BR1 by vortexing and further purification of RNA was done following the manufacturer's protocol with on-column DNase digestion. Quality of the purified RNA from was verified on an Agilent® 2100 Bioanalyzer (Agilent Technologies, Palo Alto, CA); RNA concentrations were determined using a NanoDrop® ND-1000 spectrophotometer (NanoDrop Technologies, Wilmington, DE).

Total RNA from six SCD patients and four healthy controls were used for detailed analysis on RNA-seq and exon array platforms. Data analysis was carried out on 6 SCD and 3 healthy controls after removing a control sample which was identified to be an outlier based on principal component analysis (PCA) of the transformed data from RNA-seq.

### Depletion of globin transcripts

Globin mRNA was depleted from a portion of each total RNA sample isolated from PAXgene tubes using the GLOBINclear™-Human kit (Ambion, Austin, TX). In brief 2 μg of total RNA from human whole blood was mixed with a biotinylated Capture Oligo Mix in hybridization buffer. The mixture was incubated for 15 minutes to allow the biotinylated oligonucleotides to hybridize with the globin mRNA species. Streptavidin magnetic beads were then added, to capture the globin mRNA and the magnetic beads were then pulled to the side of the tube with a magnet and the RNA, depleted of the globin mRNA, was transferred to a fresh tube. The RNA was further purified using a rapid magnetic bead-based purification method [17].

### Preparation of biotinylated cDNA targets for exon array hybridizations in GCAS

50 ng RNA samples were amplified using the WT-Ovation™ Pico RNA Amplification System (NuGEN, San Carlos, CA) in a 3 step process as recommended by the manufacturer. All processes were performed in an automated manner using the genechip array station (GCAS). In brief, first strand cDNA was prepared using a unique first strand DNA/RNA chimeric primer mix and reverse transcriptase. In the second step, DNA/RNA Heteroduplex Double Stranded cDNA was generated which serves as the substrate for SPIA amplification - a linear isothermal DNA amplification process developed by NuGEN. RNase H degrades the RNA in the DNA/RNA heteroduplex at the 5´ end of the first cDNA strand exposing part of the unique priming site for the initiation of the next round of cDNA synthesis. The process of SPIA DNA/RNA primer binding, DNA replication, strand displacement and RNA cleavage is repeated, resulting in rapid accumulation of SPIA cDNA. Following amplification and purification, 3 μg of the amplified cDNA were processed with the WT-Ovation Exon Module to produce sense strand ST-cDNA. Following purification and quantitation, 5 μg ST-cDNA was fragmented and labeled with the FL-Ovation™ cDNA Biotin Module using a proprietary two-step fragmentation and labeling process. The first step is a combined chemical and enzymatic fragmentation process that yields single-stranded cDNA products in the 50 to 100 base range. In the second step, this fragmented product is labeled via enzymatic attachment of a biotin-labeled nucleotide to the 3-hydroxyl end of the fragmented cDNA generated in the first step. Hybridization, washing and laser scanning of Affymetrix Human Exon 1.0 ST microarrays were performed according to the manufacturer’s protocol (Affymetrix, Santa Clara, CA). Hybridization was performed at 45 °C overnight, followed by washing and staining using FS450 fluidics station. Scanning was carried out using the 7 G GCS3000 scanner.

### Microarray data collection and annotation

Exon-level core RMA-sketch intensity values for each of the chips were collected using Affymetrix Expression Console (EC) Software (Affymetrix, Santa Clara, CA). The 284,258 core probesets were annotated using the Affymetrix annotation file from Netaffx (http://www.netaffx.com, HuEx-1_0-st-v2.na29.hg18.probeset.csv).

### Analysis of exon arrays

Gene level intensity values were obtained by taking the average RMA values over probesets for each transcript cluster. A two sample *t*-test (SCD N_1_ = 6 and control N_2_ = 3) was computed on 9 samples in order to determine differential gene expression between SCD and controls. Microarray RMA values for each transcript cluster for each of 9 samples were submitted to a Principal Component Analysis in order to detect possible outliers. Alternative splicing analysis was computed using the ExonANOVA available from software developed by two of the authors, J.B and P.J.M (http://affylims.cit.nih.gov/MSCLtoolbox). The ExonANOVA model fits the following formula

(1) $$y_{\mathrm{ijk}}=\mu +A_{i}+AC_{\mathrm{ik}}+C_{k}+\beta_{j\left(i\right)}+\epsilon_{\mathrm{ijk}}$$

to the data.

In the above formula, ***y***_***ijk***_ is the log 2 expression intensity, ***i*** is treatment, ***j*** is replicate within treatment and ***k*** indexes exons. The ***μ*** is the mean and the two fixed factors are the treatment effect ***(A)*** and exon effect ***(C).*** The random factor ***(β)*** is the sample within treatment effect and (**ϵ)** is the error. The fixed interaction between treatment and exon (AC) models the alternative splicing event. In this study, the treatment effect is sickle cell or control. The significance of a detected alternatively spliced event is denoted p-AC. Alternatively spliced events are declared if p-AC < = 10^-8 and the maximum absolute interaction effect (max_ik_|ACik|) is greater than or equal to 2. A p-AC < = 10^-8 corresponds to less than 1% false discovery rate (FDR) using the method of Benjamini and Hochberg [18].

### Library construction for RNA-seq

High quality total RNA at 1.5 μg was used for analysis on the Illumina GAII analyzer on six SCD samples and four healthy controls. cDNA library preparation and sequencing reactions were carried out using Illumina library prep, clustering and sequencing reagents following the manufacturer's recommendations (http://www.illumina.com). Briefly, mRNAs were purified using poly-T oligo-attached magnetic beads and then fragmented. The first and the second strand cDNAs were synthesized and end repaired. Adaptors were ligated after adenylation at the 3'-ends. After gel purification, cDNA templates were enriched by PCR. cDNA libraries were validated using a High Sensitivity Chip on the Agilent2100 Bioanalyzer™ (Agilent Technologies, Palo Alto, CA). The samples were clustered on a flow cell using the cBOT. After clustering, the samples were loaded on the Illumina GA-II machine. The samples were sequenced using a single lane with 36 cycles. Initial base calling and quality filtering of the Illumina GA-II image data were performed using the default parameters of the Illumina GA Pipeline GERALD stage (http://www.illumina.com).

### Mapping and analysis of RNA-seq data

The raw data Fastq sequence files obtained from GAII were mapped to the human genome (build HG18) to get genomic addresses using Bowtie/Tophat [19] allowing up to two mismatches. Reads that mapped to more than 10 locations were discarded. We obtained ~15.1 million reads per sample. We mapped reads both to exons of known RefSeq transcripts (human genome build 18) and to Affymetrix probe selection region coordinates. Reads mapped to Refseq exons and to Affymetrix probeset selection regions were counted using the CoverageBed method in BedTools [20]. Reads were counted for exons within each RefSeq transcript. In order to compare RNA-seq data fairly to the Exon microarray, we counted reads mapped to each probeset selection region (or probeset) within each exon***.***

Transcript cluster level reads were counted per probeset within each transcript cluster. Very low count transcript clusters (fewer than 6 samples with 6 or more counts) were ignored. This filtered out a total of 7,146 transcript clusters leaving 11,562 for further statistical analysis. In order to normalize the data, transcript cluster counts were divided by the median transcript cluster count for that sample, and logarithm base 10 transformed, yielding transformed, normalized counts. After principal component analysis (PCA) of the transformed data, one outlier, a control sample was detected and disregarded from further analysis leaving 9 samples. A two sample *t*-test (sickle cell N_1_ = 6, control N_2_ = 3) was used on the normalized, transformed data to test for differential expression. Alternative splicing analysis was computed using the ExonANOVA as with the microarrays data. A conservative and reasonable background limit at 4.5 RMA units was applied for Exon arrays and 1.0 in RPKM units as recommended by Mortazavi et al. [21] was applied for RNA_seq. The RMA level of 4.5 is slightly above the median RMA for detected exons (Affymetrix DABG value p < 0.01, Affymetrix’s recommendation for a conservative detection of exons).

In order to identify novel transcription events, we counted reads mapping to each 200 base pair region of the genome. Only populated bins (5 or more samples had 6 or more reads) bins were considered further. This filter retained 187,764 bins for analysis. We disregarded bins that fell within annotated RefSeq exons. The remaining 48,462 bins, describe novel, unannotated transcription events. To normalize these data, counts were divided by the 90th percentile of counts for that sample and base 10 logarithm transformed. p-values were required to be 0.0005 or less (corresponding to FDR < 0.15), and the fold-change was required to be 2-fold or greater. If differential expression was found, it was classified as a novel transcript.

### Real time Q-PCR analysis

First-strand cDNA was synthesized using 500 ng of RNA and random primers in a 20 μl reverse transcriptase reaction mixture using Invitrogen’s Superscript cDNA synthesis kit (Invitrogen, Carlsbad, CA) following the manufacturer’s directions. Quantitative real-time PCR assays were carried out on 11 differentially expressed genes from both the platforms with the use of gene-specific double fluorescently labeled probes in a 7900 Sequence Detector (PE Applied Biosystems, Norwalk, CT). Probes and primers were obtained from Applied Biosystems. In brief, PCR amplification was performed in a 384 well plate with a 20-μl reaction mixture containing 300 nm of each primer, 200 nm probe, 200 nm dNTP in 1x real time PCR buffer and passive reference (ROX) fluorochrome. The thermal cycling conditions were 2 min at 50° C and 10 min at 95° C, followed by 40 cycles of 15 sec denaturation at 95° C and 1 min annealing and extension at 60° C. Samples were analyzed in duplicate and the Ct values obtained were normalized to the housekeeping gene ß actin. The comparative CT (ΔΔ CT) method [22] which compares the differences in CT values between groups was used to achieve the relative fold change in gene expression between SCD and Controls.

## Results

### Principal component analysis

**Figure 1:**
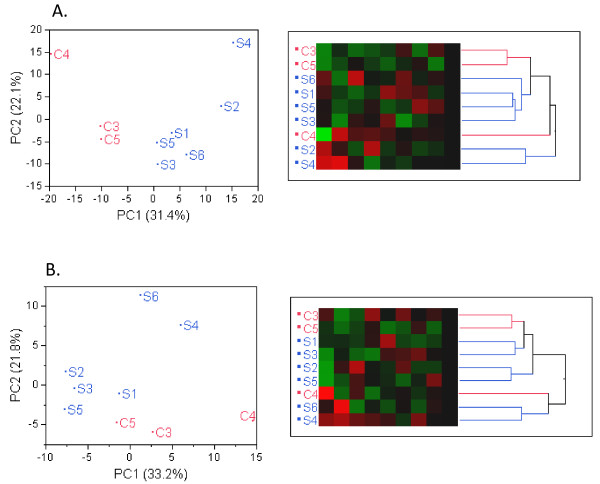
**Principal component analysis and hierarchical clustering (A) RNA-seq data. **Principal Component 1 (PC1, x-axis) represents 31.4% and PC2 (y-axis) represents 22.1% of total variation in the data. Hierarchical cluster of 9 samples with heatmap representing all 9 PCs in left-to-right order. (**B**). Exon array data. PC1 vs. PC2 on Exon array data together representing 33.2% and 21.8% of the data variability. Hierarchical clustering shows similarities to that in A, e.g. sample C4 departs strongly from remaining data, samples C3, C5 appear to be neighbors in both data sets.

Principal component analysis was first used to identify outliers within the SCD and healthy controls groups. Figure 1A for RNA-seq data showed a clear segregation and clustering of SCD and healthy controls. Similarly with the Exon array analysis on the same set of samples, a clear separation of SCD patients and healthy controls was observed as depicted in Figure 1B. The first principal component (PC1) accounted for 31% of total variability in the RNA-seq data and 33% of total variability of Exon array data, and also fully separated the sickle cell group from the controls. Sample S6 displayed the largest value for the Affymetrix QC parameter all_probesets_rle_mean, a measure of hybridization quality, where larger values indicate lesser quality. This fact may explain the divergence of patterns observed in the two dendrograms

### Evaluation of dynamic range, within platform reproducibility - coefficient of variation and sensitivity

**Figure 2:**
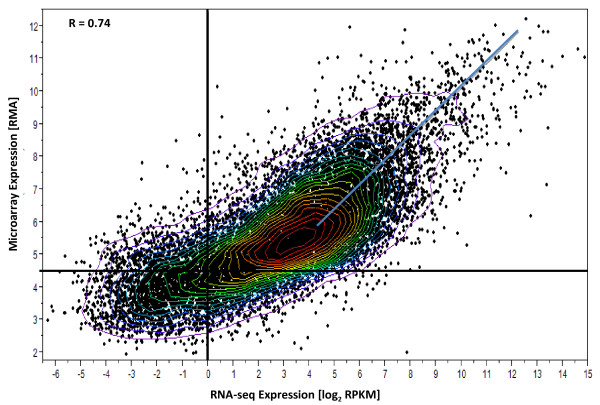
**Comparison of Gene Expression Measurements by Two Methods. Gene Microarray expression level (RMA) vs. RNA-seq expression level (log2 RPKM) for subject C3.** Both axes are expressed in base 2 logarithmic scale. The dynamic range (ratio of largest observable value to apparent background value) of the RNA-seq data is clearly larger than that of Exon array data. Bivariate density contours indicate a strong but nonlinear correlation between the two measurements. The two methods yield nearly proportional results above the median expression levels (Blue line). Solid Black lines are detection limits for microarray (RMA = 4.5) and RNA-seq (log2RPKM =0). Refer Methods for the description of detection limits.

To assess the dynamic range (the ratio of the largest observable value to the background expression level (over all genes) of each platform, a scatter plot (Figure 2) was constructed using the results on control samples for each method. The base 10 logarithm of the RPKM values from RNA-seq (normalized for gene length) is plotted versus the base 10 logarithm of signal intensity values for Exon array. As can be seen from the figure, RNA-seq shows a larger dynamic range of expression when compared to the Exon array and the magnitude of this increased dynamic range varied from 4 to 16 fold according to the expression levels of differentially expressed genes.

**Figure 3:**
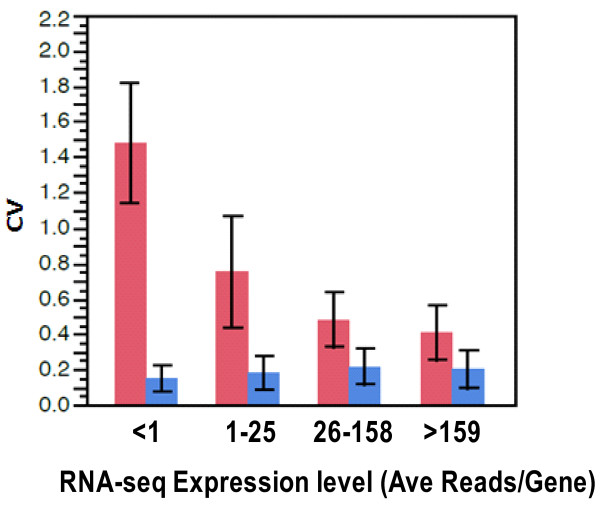
**Coefficient of Variation (CV) versus expression level for microarray and RNA-seq.** RNA-seq expression level is grouped inot 4 bins according to RNA-seq average number of reads per gene lesser than1, 1–25, 26–158, 159 or higher. CV is calculated as sample standard deviation of expression level within group (SCD and control), pooled and dvidied by mean expression level for RNA-seq (Red). For microarray (Blue), the expression values (RMA units) are first divided by ln (2) = 0.693 to convert them to a natural log scale. Then the CV is calculated as the pooled standard deviation of natural log of the expression levels.

The technical reproducibility and coefficient of variation of the array and RNA-seq platforms at both the gene and exon levels was examined using the mean expression levels over all samples. The pooled coefficient of variation calculated over all samples were broken up into 4 bins (see Figure 3 legend) Figure 3 clearly shows that the coefficient of variation for exon array is much lower than that for RNA-seq and is also independent of the number of counts for each transcript suggesting that technical variability within group is higher in the RNA-seq platform than the arrays, especially for genes with low-expression thereby demonstrating the potential advantage of microarray in cases where the RNA-seq counts are very small. The RNA-seq CV drops to 40% for highly expressed genes while for the exon array the CV rises slightly, to about 20% . This difference is partly a consequence of the extended dynamic range for RNA-seq (or equivalently, the compressed dynamic range observed in microarray).

With the expectation that deeper sequencing and large amounts of starting material are needed to adequately cover low abundance transcripts, we tested the sensitivity of each of the platforms by examining the expression of transcripts above background. With the application of conservative and reasonable background limits at 4.5 RMA units for Exon arrays and 1.0 in RPKM units for RNA_seq to one sample for a control subject C3 (Figure 2), we were able to detect 6% more transcripts (12,310/11,662 = 1.06) above background in exon arrays than in RNA-seq.

### Differentially expressed genes in SCD

**Figure 4:**
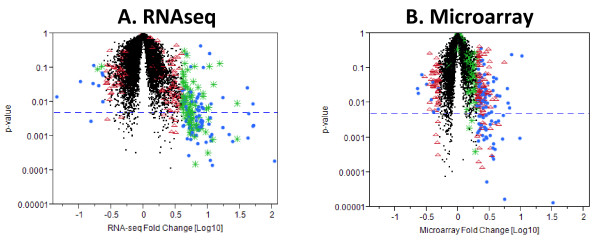
**Volcano plots for RNA-seq and Exon array. (A). p-value vs. log10 fold change (SCD vs. control) for RNA-seq data.(B).** p-value vs. log10 fold change for Affymetrix human Exon array data. Points in the lower right and lower left hand corners of the plots represent transcript clusters that are significant and differentially expressed. ●Blue circles represent transcripts with a FC greater or equal to 4 in RNA-seq and Δ Red triangles represent FC greater or equal to 2 in Exon arrays. *Green asterisks represent transcripts with a FC greater than or equal to 4 on RNA-seq only.

**Figure 5:**
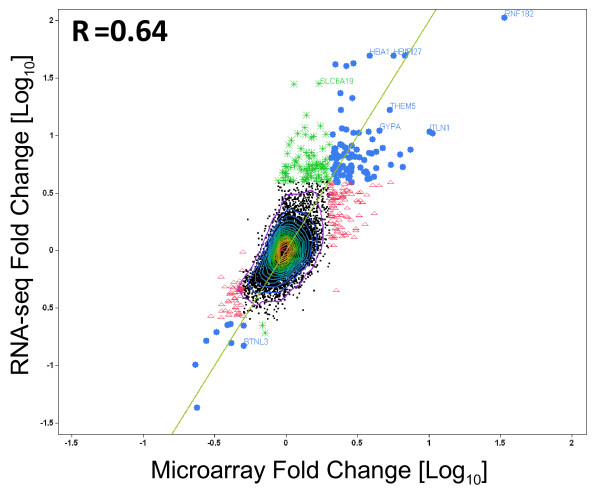
**Fold change for RNA-seq vs. fold change for Exon array (SCD vs Control).** A total of 331 transcript clusters are highlighted in the figure. The 96 blue circles ● represent transcripts with a FC greater than or equal to 4 in RNA-seq and a FC greater than or equal to 2 in microarray. The 151 red triangles ▴ represent transcripts with a fold change greater than or equal to 2 on microarray only. The 84 green asterisks * represent transcripts with a fold change greater than or equal to 4 in RNA-seq only. Correlation coefficient, R = 0.64. Genes showing greater than 4 fold change in expression levels were selected as differentially expressed in RNA-seq and genes showing greater than 2 fold change in expression levels were selected as differentially expressed in microarrays.

Global gene level expression analysis for each platform is shown in volcano plots (Figure 4). The fold changes from RNA-seq have generally larger magnitude than those from the arrays, with RNA-seq values ranging up to 100 fold, while with microarrays, we observed fold changes up to 31 fold. The relative magnitude of log fold changes can be clearly observed in Figure 4 and Figure 5, with RNA-seq reporting generally twice the log fold change compared with Exon array. In order to perform an unbiased comparative analysis of the two platforms and knowing that the microarrays have a compressed fold change, we chose two different conservative filters to select differentially expressed transcripts. We selected transcripts using different criteria for each method, specifically, requiring at least a 2-fold change in microarray data or a greater than 4-fold change in RNA-seq data. Altogether, 331 transcripts were found to be differentially expressed one or the other method, by these criteria. Many of these genes fell into pathways related to SCD including inflammatory response, oxidoreductase pathways, stress response, cell signaling and apoptosis (Table 1).

**Table 1:** Selected Differentially Expressed Genes Grouped by Pathways of Interest

**Genes**	**Symbol**	**Gene Title**	**FC** **RNA-seq**	**FC MA**
***Apoptosis/cell cycle regulation***				
NM_005581	BCAM	basal cell adhesion molecule	12.27	1.73
NM_138578	BCL2L1	BCL2-like 1	5.44	1.45
NM_198892	BMP2K	BMP2 inducible kinase	4.09	1.93
NM_004331	BNIP3L	BCL2	4.98	1.37
NM_014326	DAPK2	death-associated protein kinase 2	0.33	0.48
NM_001923	DDB1	damage-specific DNA binding protein 1, 127 kDa	2.82	2.23
NM_001122665	DDX3Y	DEAD (Asp-Glu-Ala-Asp) box polypeptide 3, Y	1.29	3.00
NM_017631	DDX60	DEAD (Asp-Glu-Ala-Asp) box polypeptide 60	1.51	2.25
NM_019030	DHX29	DEAH (Asp-Glu-Ala-His) box polypeptide 29	1.29	2.07
NM_024940	DOCK5	dedicator of cytokinesis 5	0.43	0.50
NM_004864	GDF15	growth differentiation factor 15	4.77	0.99
NM_002094	GSPT1	G1 to S phase transition 1	4.03	1.34
***Cell-signaling***				
NM_017436	A4GALT	alpha 1,4-galactosyltransferase	4.70	0.98
NR_024080	ACP1	acid phosphatase 1, soluble	2.79	2.41
NM_015256	ACSL6	acyl-CoA synthetase long-chain family member 6	6.96	2.24
NM_005622	ACSM3	acyl-CoA synthetase medium-chain family member 3	3.90	2.72
NM_001629	ALOX5AP	arachidonate 5-lipoxygenase-activating protein	0.39	0.48
NM_020980	AQP9	aquaporin 9	0.48	0.37
NM_015313	ARHGEF12	Rho guanine nucleotide exchange factor (GEF) 12	3.42	2.49
NM_015994	ATP6V1D	ATPase, H + transporting, V1 subunit D	1.54	2.21
NM_199186	BPGM	2,3-bisphosphoglycerate mutase	11.03	2.89
NM_020850	RANBP10	RAN binding protein 10	5.70	2.31
NM_001145657	RAP1GAP	RAP1 GTPase activating protein	42.05	2.58
NM_014737	RASSF2	Ras association (RalGDS	0.45	0.47
NM_002923	RGS2	regulator of G-protein signaling 2, 24 kDa	0.32	0.44
NM_000062	SERPING1	serpin peptidase inhibitor, clade G (C1 inhibitor)	3.58	2.03
NM_001122752	SERPINI1	serpin peptidase inhibitor, clade I (neuroserpin), 1	1.47	3.07
NM_007111	TFDP1	transcription factor Dp-1	5.69	2.45
NM_003227	TFR2	transferrin receptor 2	5.67	1.42
NM_173804	TMEM86B	transmembrane protein 86B	5.22	1.39
NM_015049	TRAK2	trafficking protein, kinesin binding 2	5.51	3.24
NM_022066	UBE2O	ubiquitin-conjugating enzyme E2O	7.13	2.83
***Oxidative stress/antioxidants/Stress Response***				
NM_000032	ALAS2	aminolevulinate, delta-, synthase 2	4.36	1.66
NM_001128829	CA1	carbonic anhydrase I	10.68	2.07
NM_000067	CA2	carbonic anhydrase II	5.03	3.45
NM_016417	GLRX5	glutaredoxin 5	5.24	1.59
NM_201397	GPX1	glutathione peroxidase 1	8.02	2.16
NM_001145260	NCOA4	nuclear receptor coactivator 4	4.77	2.12
NM_002501	NFIX	nuclear factor I	5.73	1.73
NM_000274	OAT	ornithine aminotransferase (gyrate atrophy)	1.30	2.19
NM_030758	OSBP2	oxysterol binding protein 2	11.80	2.57
NM_032523	OSBPL6	oxysterol binding protein-like 6	4.70	2.45
NM_005809	PRDX2	peroxiredoxin 2	4.10	1.12
NM_006745	SC4MOL	sterol-C4-methyl oxidase-like	1.31	2.47
NM_138432	SDSL	serine dehydratase-like	6.58	1.21
NM_003944	SELENBP1	selenium binding protein 1	5.76	1.92
NM_175839	SMOX	spermine oxidase	5.09	2.78
NM_003794	SNX4	sorting nexin 4	1.43	2.29
NM_003105	SORL1	sortilin-related receptor, L(DLR class) A repeats	0.48	0.41
NM_003227	TFR2	transferrin receptor 2	5.67	1.42
NM_022648	TNS1	tensin 1	4.21	1.05
***Inflammatory Response***				
NM_005581	BCAM	basal cell adhesion molecule (Lutheran blood group)	12.27	1.73
NM_003965	CCRL2	chemokine (C-C motif) receptor-like 2	2.00	2.28
NM_012120	CD2AP	CD2-associated protein	2.30	2.20
NM_001001548	CD36	CD36 molecule (thrombospondin receptor)	1.68	2.11
NM_152243	CDC42EP1	CDC42 effector protein (Rho GTPase binding) 1	8.43	1.04
NM_004344	CETN2	centrin, EF-hand protein, 2	2.85	2.46
NM_001008388	CISD2	CDGSH iron sulfur domain 2	7.29	3.91
NM_033554	HLA-DPA1	major histocompatibility complex, DP alpha 1	1.32	2.11
NM_002125	HLA-DRB5	major histocompatibility complex, class II, DR beta 5	1.96	2.09
NM_001130080	IFI27	interferon, alpha-inducible protein 27	51.57	6.64
NM_006417	IFI44	interferon-induced protein 44	3.14	3.21
NM_006820	IFI44L	interferon-induced protein 44-like	3.86	5.37
NM_001548	IFIT1	interferon-induced protein with tetratricopeptide 1	3.04	2.62
NM_000576	IL1B	interleukin 1, beta	0.43	0.41
NM_004633	IL1R2	interleukin 1 receptor, type II	0.17	0.27
NM_002182	IL1RAP	interleukin 1 receptor accessory protein	0.29	0.40
NM_002183	IL3RA	interleukin 3 receptor, alpha (low affinity)	0.52	0.49
NM_003024	ITSN1	intersectin 1 (SH3 domain protein)	6.28	1.87
NM_003189	TAL1	T-cell acute lymphocytic leukemia 1	5.37	1.76
NM_017772	TBC1D22B	TBC1 domain family, member 22B	3.78	2.82
NM_152772	TCP11L2	t-complex 11 (mouse)-like 2	3.32	2.64
***Red Cell genes***				
NM_020476	ANK1	ankyrin 1, erythrocytic	4.04	1.62
NM_152326	ANKRD9	ankyrin repeat domain 9	9.23	1.08
NM_001728	BSG	basigin (Ok blood group)	5.01	1.50
NM_016633	ERAF	erythroid associated factor	9.64	3.91
NM_001017922	ERMAP	erythroblast membrane-associated protein	3.86	2.05
NM_001012515	FECH	ferrochelatase (protoporphyria)	8.10	2.56
NM_002099	GYPA	glycophorin A (MNS blood group)	11.49	4.40
NM_002100	GYPB	glycophorin B (MNS blood group)	7.43	2.11
NM_198682	GYPE	glycophorin E	6.67	2.70
NM_000558	HBA1	hemoglobin, alpha 1	51.86	5.54
NM_000518	HBB	hemoglobin, beta	28.47	1.12
NM_000519	HBD	hemoglobin, delta	44.50	2.88
NM_005330	HBE1	hemoglobin, epsilon 1	24.48	2.34
NM_000559	HBG1	hemoglobin, gamma A	43.18	2.17
NM_001003938	HBM	hemoglobin, mu	3.75	2.04
NM_005332	HBZ	hemoglobin, zeta	8.72	2.34
NM_018437	HEMGN	hemogen	5.83	2.18
NM_000420	KEL	Kell blood group, metallo-endopeptidase	4.59	1.67
NM_006563	KLF1	Kruppel-like factor 1 (erythroid)	6.76	1.41
***Novel Genes***				
NM_007021	C10orf10	chromosome 10 open reading frame 10	10.26	1.35
NM_001009894	C12orf29	chromosome 12 open reading frame 29	1.39	2.71
NM_014059	C13orf15	chromosome 13 open reading frame 15	2.22	2.03
NM_025057	C14orf45	chromosome 14 open reading frame 45	4.54	4.02
AK303128	C17orf99	chromosome 17 open reading frame 99	5.43	1.24
BC038410	C1orf105	chromosome 1 open reading frame 105	6.00	1.16
NM_020362	C1orf128	chromosome 1 open reading frame 128	5.23	2.58
NM_015680	C2orf24	chromosome 2 open reading frame 24	4.06	1.75
NM_001042521	C2orf88	chromosome 2 open reading frame 88	5.41	2.19
NM_001002029	C4B	complement component 4B	4.22	1.37
NM_000715	C4BPA	complement component 4 binding protein, alpha	0.04	0.23
NM_001128424	C4orf18	chromosome 4 open reading frame 18	1.35	2.60
NM_032412	C5orf32	chromosome 5 open reading frame 32	3.04	2.12
NM_032385	C5orf4	chromosome 5 open reading frame 4	7.20	2.18
NM_052831	C6orf192	chromosome 6 open reading frame 192	2.08	2.36
NR_027330	C7orf54	chromosome 7 open reading frame 54	0.67	0.49

* Differentially expressed genes were selected if they showed greater than 4-fold change (SCD v Control) in RNAseq or greater than 2-fold change in Microarray.

**Figure 6:**
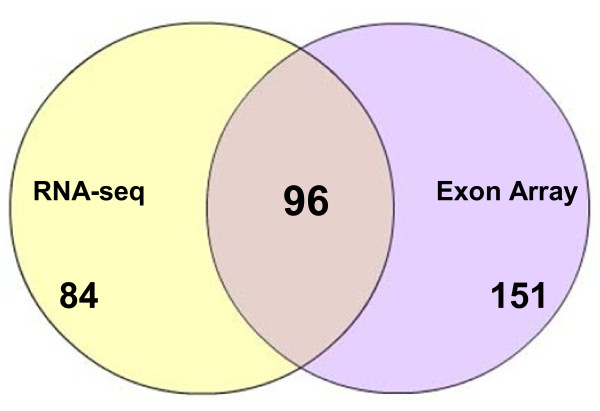
**Venn diagram showing the 331 differentially expressed genes between SCD and Healthy Controls for RNA-seq and microarray.** Gene selection Criteria for RNA-Seq -FC greater than or equal to 4; Exon array -FC greater than or equal to 2.

Of the 331 selected transcripts, 84 transcripts pass only the RNA-seq filter, 151 transcripts pass only the exon array filter and 96 transcripts pass both (Figure 6). Of the 331 transcripts, only 11 (3.3%) were discordant in their direction of change as measured by the two methods and none of these were among the 96 passing both filters. The cross platform correlation for the differential expression (Figure 5) was substantial (r = 0.64), indicating that the two methods give highly similar results overall. With the additional requirement of statistical significance p < 0.005 corresponding to FDR <24% for RNA-seq and FDR < 35% for Exon array, only 112 transcripts showed changes in one or both methods. Of these, 54 showed differential expression only in RNA-seq, 27 showed differential expression in only Exon array, and 31 showed differential expression in both methods.

Genes identified as differentially expressed in SCD were subjected to gene ontology enrichment analysis to determine their molecular functions. This analysis selected 10 highly significant functional pathways including cell cycle regulators, apoptosis, oxidative stress response, inflammation and immune response, free radical scavenging, protein modification, and hematopoiesis. (Additional file 1: Figure S1). Examination of these pathways suggests that these differentially regulated genes are consistent with the homeostatic response to known pathobiological stresses in SCD, including oxidative and hemolytic stress, vascular injury, and participation in repair. We also observed upregulated expression of several reticulocyte specific genes such as ankyrin1, erythroid associated factor, hemoglobins, nuclear associated factor, glycophorin, transferrin receptor, and selenium binding protein, as expected with prominent reticulocytosis in SCD, thereby validating the performance of the two technologies in identifying biological alterations in the sickle cell disease model.

### Effect of globin reduction on RNA-seq data quality

Currently, RNA-seq requires enrichment steps to select Poly A RNA for library construction from total RNA. Since ribosomal RNA represents over 90% of the RNA within a given cell, studies have shown that its removal increases the sensitivity to retrieve data from the remaining portion of the transcriptome. In large clinical studies where whole blood PAXgene RNA is used, there is an additional interference by the high levels of globins in whole blood RNA. This is further complicated in hematologic diseases such as sickle cell where globins account for more than 70% of mRNA.

In order to determine if globins interfere with the sequence reads and affect the sensitivity of transcriptome analysis on RNA-seq platform, we reduced the globins on one sickle cell patient sample and compared the globin reduced and non-reduced samples on RNA-seq. Scatter plot analysis of the normalized transcript read counts for these two samples showed a high correlation (R = 0.93, Additional file 2: Figure S2). This suggests that the globin transcripts in the sickle cell sample do not affect the sequence reads and do not introduce much bias in the analysis.

### Validation by QPCR analysis

**Figure 7:**
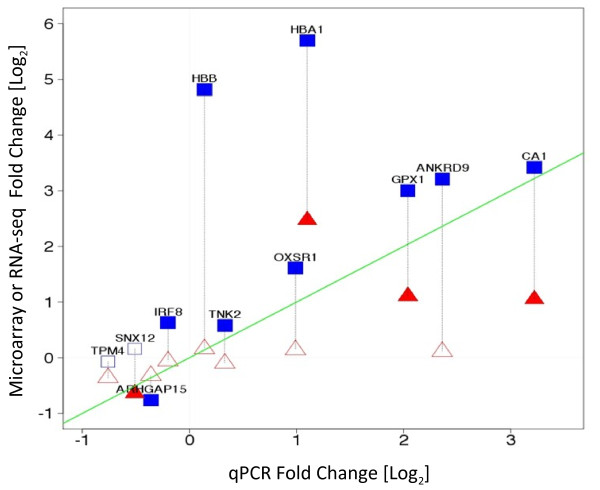
**Validation by QPCR - Log2 expression fold change (SCD vs. Control) measured by microarray (Red) or RNA-seq (Blue) vs. qPCR on selected genes.** Closed symbols represent significant changes, open signals are not significant. The green line is the line of identity. Symbols closer to the line of identity are in better agreement with QPCR. ▴Significantly differentially expressed genes by microarray, ■Significantly differentially expressed genes by RNA-seq; Δ No significance in microarray; □No significance in RNA-seq.

Taqman analysis was used to validate 11 selected differentially expressed genes identified by one or both the microarray and RNA-seq platforms. The concordance of each platform with QPCR analysis was measured by Pearson’s correlation on the fold changes. A good degree of correlation was observed for most of the genes across the three platforms (Additional file 3: Table S2). RNA-seq and QPCR showed a correlation R = 0.6; while QPCR and Exon array revealed a correlation of R = 0.58. The line of identity shown in Figure 7 illustrates the concordance between the platforms. The most highly correlated genes between the platforms are those that lie closest to the line of identity in the figure. Genes *IRF8, SNX12*, and *TPM 4* showed directional discrepancy between microarray and RNA-seq, however QPCR analysis corroborated the microarray data for those genes. Conversely, QPCR analysis of gene *TNK2,* which was found to be up-regulated by RNA-seq and down regulated by Exon array, corroborated the RNA-seq analysis.

### Detection of alternatively spliced exons

Using ExonAnova analysis and filtering criteria as set forth in the Methods section, we were able to identify 16 genes displaying alternative splicing using the RNA-seq platform including *ATF6B, BCL6, CARM1, CCNDBP1, COX4I1, DCTN2, HPS1, INPP5D, INSIG1, NUDT, NUDT4P1, RHCE, RHD, TNXA, TNXB,* and *UNC13D* as shown in Table 2. Exon array, on the other hand was able to identify only *HBA1* as a significantly alternatively spliced gene while the other genes did not meet the statistical filter for splicing*.*

**Table 2:** Highly Significant Alternatively Spliced Genes

**Symbol**	**GeneTitle**	**Molecular Functions**	**RNAseq**	**MA**
			**Splicing Index**	
ATF6B*	activating transcription factor 6 beta	Cell Death/Immune Response	0.49*	0.17
BCL6*	B-cell CLL	Apoptosis/Immune Response	0.68*	0.07
CARM*	coactivator-associated arginine methyltransferase 1	Cell Development/Differentiation	0.40*	0.12
CCND*	cyclin D-type binding-protein 1	Cell Cycle	0.63*	0.13
COX4I*	cytochrome c oxidase subunit IV iso1	Cellular Development/Compromise	0.89*	0.04
DCTN*	dynactin 2 (p50)	Cellular Movement	0.47*	0.08
HPS1*	Hermansky-Pudlak syndrome 1	Inflammatory Response; Cell Signaling	0.39*	0.15
INPP5*	inositol polyphosphate-5-phosphatase	Cell-Cell Signaling	0.49*	0.07
INSIG1*	insulin induced gene 1	Lipid metabolism, Molecular Transport	0.52*	0.09
NUDT*	nudix type motif 4	Cell signaling; Hematopoiesis	0.37*	0.16
NUDT*	nudix motif 4 pseudogene	Cell signaling; Hematopoiesis	0.37*	0.16
RHCE*	Rh blood group, CcEe antigens	Agglutination of red blood cells	0.54*	0.18
RHD*	Rh blood group, D antigen	Agglutination of red blood cells	0.54*	0.18
TNXA*	tenascin XA (pseudogene)	Cellular Assembly	0.49*	0.17
TNXB*	tenascin XB	Cellular Assembly	0.49*	0.17
UNC13*	unc-13 homolog D	Cell Signaling	0.76*	0.10
HBA1**	**hemoglobin, alpha 1**	**Transport of oxygen**	**0.09**	0.57**

* Selected genes from RNA-Seq with P < 0.00000001.

** Selected Genes from Microarray with P < 0.00000001.

### Discovery of novel transcripts to be differentially expressed in SCD

RNA-seq technology is capable of discovering novel transcripts and novel isoforms as it is not constrained to measure only pre-defined transcripts, as is the microarray. Instead of mapping reads to known transcripts, we mapped reads to the entire genome, and collected them into 200 base pair bins (see Methods) to identify such novel transcripts. We identified 86 novel regions that manifest a significant (p < 0.005; 15% FDR), greater than 2-fold change between sickle cell disease and control groups (Table 3).

**Table 3:** Highly Significant Novel Differentially Expressed 200 bp Regions

**Chromosome**	**Start-Base −1**	**End- Base 200**	**Fold Change**	**p Value**
chr1	203465800	203465999	18.69	0.00005
chr1	246094400	246094599	12.84	0.00018
chr1	144990000	144990199	4.96	0.00016
chr1	209819000	209819199	4.93	0.00022
chr1	554400	554599	0.44	0.00050
chr1	91770600	91770799	0.43	0.00030
chr1	157083400	157083599	0.33	0.00034
chr1	159838000	159838199	0.26	0.00025
chr2	177785200	177785399	9.45	0.00049
chr2	128992200	128992399	5.46	0.00038
chr2	91411400	91411599	3.38	0.00037
chr2	175293200	175293399	0.50	0.00037
chr2	207652800	207652999	0.35	0.00012
chr3	76567600	76567799	0.49	0.00029
chr4	154075400	154075599	10.11	0.00029
chr4	38368600	38368799	9.62	0.00004
chr4	154075800	154075999	7.75	0.00005
chr4	146763600	146763799	4.91	0.00035
chr4	146765600	146765799	4.82	0.00018
chr4	111338400	111338599	4.66	0.00028
chr4	146516600	146516799	0.39	0.00014
chr4	26043400	26043599	0.38	0.00045
chr5	176439200	176439399	5.84	0.00048
chr5	138854200	138854399	5.30	0.00014
chr5	138856400	138856599	3.34	0.00006
chr5	177142200	177142399	3.10	0.00046
chr5	43623000	43623199	0.46	0.00018
chr5	99410000	99410199	0.26	0.00010
chr6	53038000	53038199	10.59	0.00015
chr6	151297600	151297799	5.46	0.00021
chr6	28212400	28212599	2.43	0.00004
chr7	55681200	55681399	3.73	0.00047
chr7	5567000	5567199	0.48	0.00041
chr7	139365000	139365199	0.35	0.00034
chr8	41761600	41761799	8.49	0.00001
chr8	41762200	41762399	6.42	0.00046
chr8	130922200	130922399	0.34	0.00033
chr9	35101200	35101399	22.26	0.00009
chr9	35101400	35101599	18.75	0.00019
chr9	5101000	5101199	3.04	0.00020
chr9	79521600	79521799	0.38	0.00020
chr9	79523600	79523799	0.36	0.00014
chr10	13766600	13766799	12.54	0.00002
chr10	91112400	91112599	11.27	0.00042
chr10	75248600	75248799	0.37	0.00000
chr10	81901200	81901399	0.35	0.00036
chr11	5225800	5225999	24.75	0.00001
chr11	94542200	94542399	2.35	0.00002
chr11	117571400	117571599	0.37	0.00034
chr11	6193200	6193399	0.34	0.00012
chr12	111304400	111304599	9.16	0.00022
chr12	105247200	105247399	7.71	0.00033
chr12	88443200	88443399	3.83	0.00014
chr12	62331800	62331999	0.47	0.00032
chr13	74302000	74302199	7.60	0.00014
chr13	18137800	18137999	3.88	0.00013
chr14	65417400	65417599	6.12	0.00029
chr14	65417000	65417199	5.15	0.00033
chr14	52176600	52176799	0.36	0.00046
chr15	72689200	72689399	11.91	0.00011
chr15	72678200	72678399	6.26	0.00018
chr15	72688400	72688599	6.17	0.00014
chr15	72687200	72687399	5.89	0.00028
chr15	79393000	79393199	0.43	0.00002
chr17	39736800	39736999	9.65	0.00025
chr17	39632800	39632999	3.77	0.00047
chr17	35692200	35692399	0.43	0.00047
chr18	14383400	14383599	3.15	0.00016
chr19	51317000	51317199	7.49	0.00019
chr19	56938000	56938199	0.31	0.00035
chr20	55405400	55405599	13.85	0.00012
chr20	30909600	30909799	0.44	0.00014
chr20	1403000	1403199	0.29	0.00036
chr21	14058800	14058999	4.92	0.00006
chr22	29607600	29607799	12.14	0.00048
chr22	35263200	35263399	6.35	0.00047
chr22	43511000	43511199	2.97	0.00018
chr22	20569800	20569999	0.45	0.00029
chr22	22898800	22898999	0.34	0.00010
chrM	12600	12799	0.44	0.00026
chrX	55067400	55067599	5.80	0.00033
chrX	125434800	125434999	0.50	0.00005
chrX	143984400	143984599	0.41	0.00005
chrX	1389000	1389199	0.34	0.00037
chrY	3611200	3611399	8.93	0.00011
chrY	1389000	1389199	0.34	0.00037

**Figure 8:**
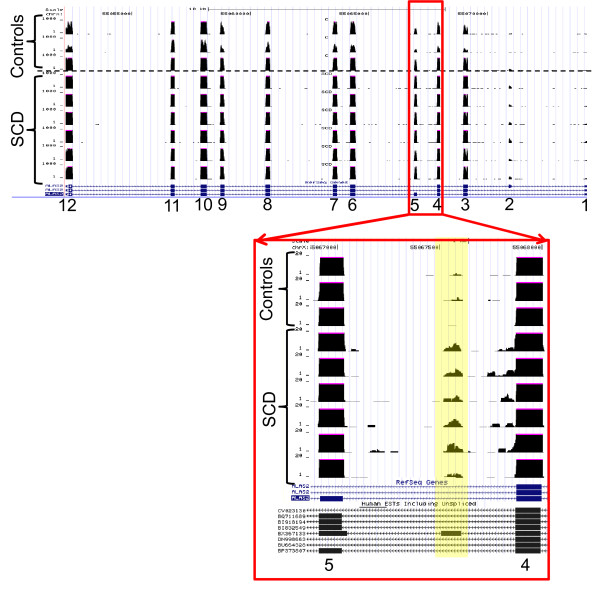
**Coverage Plot of RNA-seq data for*ALAS*2 gene RNA-seq reads for*ALAS*2 gene are shown in genomic context (chrX:55,051,744-55,074,222).** An apparently novel exon dubbed 4a, between exons 4 and 5 is expressed significantly more in SCD compared with controls (p = 0.0003). This exon has been previously observed as human EST BX367133, in a clone derived from T cells. The inset shows the region bounded by exons 4 and 5 with the coverage range expanded and truncated to 20 for each track.

One novel region was found within the differentially expressed ALAS2 gene. Figure 8A shows the high expression levels detected for each exon of that gene. Based on the known exons, the computed expression change was 4 fold. One 200 bp region (Table 3, chrX:55067400–55067599) falls between exons 4 and 5, and showed a significant (P < 0.0003), 6 fold expression change, but with expression levels that are nearly invisible in the context of the surrounding exons (Figure 8). Figure 8 and the Additional file 4: Figure S3 (zoomedin exon 4A) show that expression is in fact present in this region for the SCD patients but nearly completely absent in controls. Curiously, expression in this apparently novel region has been previously observed in a single EST derived from T-cells (EST BX367133). It is likely that this represents a rarely used, alternative exon for the ALAS2 gene with greater expression in SCD (6-fold vs 2-fold). Similarly, 200 bp bin analysis showed several differentially expressed regions in chromosomes X, Y, M, 1–12, 19, 20 and 22 and these regions mapped to mitochondrial genes with some regions representing psuedogenes. These results are shown in Table 3. Additional file 5: Table S1 shows few examples of the alignment of sequences by BLAST.

### Analysis for sequence variation in expressed transcripts

**Figure 9:**
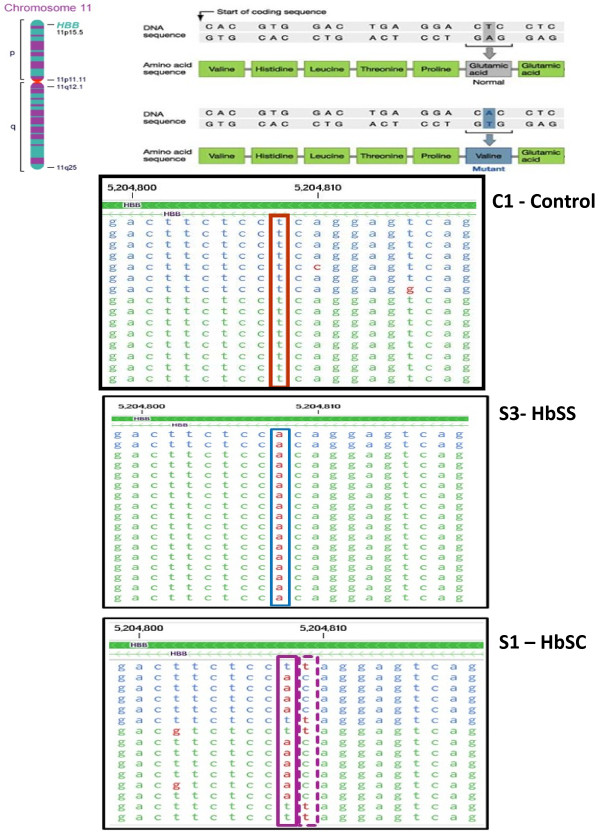
**Analysis of sequence variants in the expressed hemoglobin transcript in a Healthy Control – (C1), and Homozygous (S3-HbSS) and Heterozygous (S1-HbSC) Sickle Cell Patients Observed sequences of HBB (hemoglobin B) gene in the region including the known sickle cell mutation, which causes a substituion of valine (coded by CAC) for glutamic acid (coded by CTC).** The box for the reads from sample C1 - control, show the observed sequences (on the coding strand, but in reversed order) and are consistently T at the mutation position. The box for sample S3 - HbSS shows the consistent substitution of A at this same position. The box for sample S1 - HbSC show approximately 50% substitution of A for T at this position, and an additional mutation at the neighboring postion C- > T. This sample was revealed to be from a compound heterozygous hemoglobin SC patient.

RNA-seq is an attractive method that enables the identification of sequence variations in expressed transcripts. To illustrate the feasibility of identifying sequence variation in expressed genes, we visualized the sequence of reads from the beta globin gene (DNAnexus, https://dnanexus.com/) for SCD and control subjects in the neighborhood of the known single mutation driving sickle cell disease. In SCD, glutamic acid (coded by CTC) is replaced by Valine (coded by CAC). Figure 9 clearly identifies this mutation in the sickle cell sample as compared to the controls. Interestingly, one of our sickle cell patients (S1) was a compound heterozygous hemoglobin SC patient and the heterozygosity in this patient is clearly seen in Figure 9. This demonstrates the ability of the RNA-seq technology to identify sequence variation in the expressed transcripts that would not be detected in the microarray analysis.

## Discussion

Whole transcriptome sequencing (RNA-seq) is a powerful transcriptional profiling technology using next generation sequencing platforms [4,23-25] and has signaled a new age in clinical genomics. Several recent studies have indicated that RNA-seq will be more useful than the current microarray technology due to the increased dynamic range of signal of sequencing [4,26-28] and its ability to identify the exact location of transcription boundaries at single base resolution. RNA-seq also provides the sequence information needed to identify single nucleotide variants, map variant transcription start sites, and detect novel transcript splicing. These features make RNA-seq particularly useful for studying complex transcriptomes, such as those found in the clinical blood samples.

Although attractive, clinical application of RNA-seq is feasible only if the tool can demonstrate high specificity, sensitivity and reproducibility with limited amount of starting material. Although current technology for transcriptome sequencing requires at least 100 ng total RNA (tens of thousands of cell equivalents), along with additional enrichment steps to select for poly(A) + RNA and/or to reduce the content of ribosomal RNA (rRNA) prior to NGS library construction, to minimize the loss of input material researchers tend to start with a minimum of 1 microgram total RNA. The utility of RNA-seq data generated is believed to be sensitive to read length, mapping and assembly of reads and statistical and computational challenges. However, with the current availability of substantially improved mapping software, these challenges are expected to be well tackled. Microarrays, on the other hand, are known to suffer from reduced dynamic range of signals due to saturation biases and high background, and non-specific or cross-hybridization resulting in false-positive signals, especially for transcripts that have low expression levels.

Considering these challenges inherent to each of the high throughput platforms, we undertook this comparative study in an attempt to better understand the relative merits of high density exon microarrays and RNA-seq for biomarker discovery in the clinical setting. We chose the sickle cell disease because strong differential expression has been previously observed, and the phenotype of the disease is in manifested in blood, an accessible tissue for study. Sampling whole blood, a globin abundant tissue, allowed us to examine the potential interference of high abundant globin transcripts during sequencing and also to potentially discover novel genes that are associated with sickle cell disease from cell types such as nucleated red cells in addition to the conventional peripheral blood mononuclear cells. We believe that this is the first study that has compared the 2 platforms on a monogenic human disease model using easily accessible whole blood clinical specimens mimicking a large scale clinical research project.

In this study we used 50 ng of total RNA on exon arrays without any globin reduction or poly(A) + enrichment to identify differentially expressed genes and alternative splicing events in sickle cell disease. The same samples were also analyzed by RNA-seq using 1.5 micrograms of total RNA. RNA-seq analysis generated an average of 83% mappable reads from the whole blood samples after poly(A) + enrichment. The globin reduction process had insignificant effect on the mappable read count, even for sickle cells samples which have high levels of globin RNA suggetsing that it is not necessary to reduce the globins while analyzing whole blood samples by RNA-seq.

As expected, comparison of the dynamic range of the two platforms confirmed that RNA-seq has a dramatically larger range varying from 4 to 16 fold. This enticing feature of RNA-seq effectively removes the saturation biases inherent to the array platform. Examination of the technical reproducibility and coefficient of variation of the array and RNA-seq platforms at the gene and exon levels, as a function of the mean expression level, indicated that the coefficient of variation for microarray is much lower than that for RNA-seq and is also independent of the expression level for each transcript, suggesting that technical variability within group is higher in the RNA-seq platform than the arrays. A similar observation has been reported by Marioni et al. [15]. They observed extremely high CVs when the read counts were low, a domain where Poisson counting error dominates in RNA-seq. In this domain, microarray produces moderately low CV (20%), suggesting that microarray may in fact be more effective at detecting expression changes for low-abundance genes.

Our comparative analysis of detection sensitivity with material from clinical samples revealed that even with the usage of 30 times less starting material (50 ng vs 1.5 micrograms) Exon arrays could detect as many transcripts above background as in RNA-seq. It should be noted that the sequencing depth in this study (~10 million reads) is comparable with most published RNA-seq studies. Xu and others [29] from their comparative study using GG Exon arrays to RNA-seq reported that although both platforms detect similar expression changes at the gene level, the Exon array is more sensitive at the exon level and deeper sequencing is required to adequately cover low abundance transcripts [29]. It should be mentioned here that with the latest much improved sequencing instruments, it would be easier to generate ~ 80 million reads and this would substantially increase the sensitivity of detection in RNA-seq platform.

We found 331 transcripts with differentially expressed transcripts in SCD. These included genes involved in pathways related to sickle cell disease such as inflammatory response, oxidoreductase pathways, stress response, cell signaling and apoptosis. Of these 331 transcripts which showed a high degree of correlation (R = 0.64), 96 genes were identified by both the technologies. A similar observation has also been reported by correlating gene expression arrays and RNA-seq on their study on differentially expressed liver and kidney tissues [15]. Only 11 genes out of the 331 genes from the current study showed an opposite trend in differential expression in sickle cell disease, suggesting that the number of false positives was small, using either method.

Gene ontology analysis of these genes helped to classify their molecular functions into ten highly significant functional pathway such as cellular cycle regulators, apoptosis, oxidative stress response, inflammation and immune response, free radical scavenging, protein modification, and hematopoiesis. Examination of these pathways suggests that differentially regulation may be in response to oxidant and hemolytic stress, vascular injury and participation in repair and homeostasis [17,30-34]. Interestingly, GDF15 expression was upregulated, which also has been observed in thalassemia intermedia, and associated with repression of hepcidin, an important mediator of the inflammatory response on erythropoiesis [35].

We also observed upregulated expression of several reticulocyte specific genes as expected in SCD where a higher proportion of reticulocytes are observed. This finding validates the performance of both technologies in identifying alterations relevant to sickle cell disease. From a biological perspective, the whole blood expression profile provided a window into real time erythrocyte expression profiles. Insights into the transcription profile of these red blood cells may contribute greatly to our understanding of mechanism of disease, prognosis, and responses to therapeutics.

Using ExonAnova analysis on RNA-seq data, we identified 16 alternatively spliced genes. While further validation of these splice variants is needed, it is interesting to note that both *RHCE* and *RHD* are components of the important Rh antigen system on red cells. However the potential implications of altered Rh splicing in SCD is still unclear. Deficiency of *UNC13D* is known to result in defective exocytosis of cytolytic granules of cytotoxic T lymphocytes and natural killer cells, causing immune dysregulation [36]. Whether altered expression of UNC13D in SCD could contribute to the relative immune compromise of SCD may merit future investigation.

To illustrate the power of RNA-seq in detecting differential transcription not associated with known genes, we scanned the entire genome for novel differential expression focusing only on unannotated genomic regions. By doing so, we found an interesting region to include an apparently novel, minor exon between exons 4 and 5 in the *ALAS2* gene with SCD patients showing at least six times higher expression levels compared to the control subjects (p = 0.003). This could suggest alternative splicing in SCD which might serve as an *ALAS2* transcription regulator. Follow up of this suggestion would require a functional analysis of this newly identified region of *ALAS*2 but is beyond the scope of the current study but is planned for the future. ALAS2 gene expression is restricted to the erythroid lineage [37] and plays a pivotal role in heme synthesis. In addition to heme-mediated feedback inhibition of enzymatic function, ALAS-2, a member of a small family of genes is modulated by iron [38]. This ability of RNA-seq to identify regions in detail holds great promise for the future discovery of novel transcripts and biomarkers in clinical genomic studies.

Another key advantage of RNA-seq over existing technologies for transcriptomic studies is its ability to identify sequence variations in expressed transcripts. To illustrate the feasibility of identifying sequence variation in expressed genes, we focused on the known single nucleotide mutation in SCD in which glutamic acid-6 is replaced by valine (GAG replaced by GTG). We were able to successfully detect this mutation in all the sickle cell patients. Interestingly, we were also able to identify, that same mutation in heterozygous combination with a hemoglobin C beta globin variant having glutamic acid replaced by lysine (GAG replaced by AAG) in one compound heterozygous sickle cell patient, thereby demonstrating the ability of RNA-seq to reliably identify heterozygous single base mutations in the expressed transcripts.

In conclusion, our study clearly illustrates a high level of concordance between the array platform and the RNA-seq technology, and suggests that the high density Exon array still remains a powerful tool to generate meaningful data when the amount of material is limited. Although RNA-seq is still in the early stages of use in clinical studies, it has clear advantages over the array based transcriptomic methods, based on its ability to discover novel transcripts, identify sequence variants, and increased dynamic range of signals leading to increase fold change in measured expression levels. With the rapid evolution of NGS instruments and library preparation methods with multiplexing barcodes, longer read lengths and large number of paired end reads associated with reduced cost per lane is highly feasible in the near future. The use of picogram to few nanogram amounts to total RNA for RNAseq still needs to be optimized in order to capture low abundance transcripts.

We believe that the results from this study provide guidelines on the choice of tools in the form of arrays or RNA-seq for clinical transcriptomic studies using limited amount of starting material. We believe that the selection of an appropriate tool for clinical genomic studies is mostly driven by the biological question underlying the study: whether a formal hypothesis is being tested or is the study intended to better describe the complete transcriptome and discover novel transcripts. An emerging approach is to apply both RNA-seq and arrays in combination, in large scale clinical studies, where RNA-seq is used first to define the transcriptome elements associated with the disease in question, followed by high throughput and reliable screening of these elements on thousands of patient samples using the arrays. Integrating data from both microarray and RNA-seq experiments may open up new possibilities for creating meaningful informational networks which will aid our understanding of disease pathology and development of novel therapeutics.

## Competing interests

The authors declare that they have no competing interests.

## Authors’ contributions

NR designed the study, wrote the manuscript, NR, PL, KW, performed the experiments. YY helped in mapping the NGS data. JB, PJM performed data analysis and edited the manuscript. GK initiated the NIH protocol for patient recruitment, sample collection and edited the manuscript. DL and CJO funded the study and edited the manuscript. All authors have read and approved the final manuscript.

## Pre-publication history

The pre-publication history for this paper can be accessed here:

http://www.biomedcentral.com/1755-8794/5/28/prepub

## Supplementary Material

Additional file 1 **Figure S1.** Gene Ontology analysis on the differentially expressed genes. The top 13 highly significant classification/functions of genes are shown in the figure. Click here for file

Additional file 2 **Figure S2.** Effect of Globin Reduction on RNA-seq expression. Y-axis: Read counts per transcript, normalized by median for globin reduced sample; X-axis: Read counts normalized by median for same sample using standard preparation. Correlation coefficient is 0.93. Click here for file

Additional file 3 **Table S2.** Validation of few differentially expressed genes by QPCR. Click here for file

Additional file 4 **Figure S3.** Putative Exon 4A . UCSC Genome Browser view of the BAM files for each sample showing genomic region chromosome X: 55067500–5506725. The first 3 aligned tracks show the control samples, the following 5 tracks show the sickle cell disease samples. Aligned reads are Red if to the negative strands and blue if to the positive strands. The total reads per sample is: C3: 15,715,705, C4: 15,131,360, C5: 15,730,372, S6: 16,570,843, S1: 13,481,528, S2: 16,707,788, S3: 14,650,161, S4: 18,580,778 and S5: 16,460,443. S6: 16570843, S1. Click here for file

Additional file 5 **Table S1.** Complete List of differentially expressed genes (n = 331). Click here for file
