# Supplementary material for: A systematic comparison and evaluation of high density exon arrays and RNA-seq technology used to unravel the peripheral blood transcriptome of sickle cell disease
Source: BMC Med Genomics. 2012 Jun 29;5:28. doi: 10.1186/1755-8794-5-28 (PMC3428653; doi:10.1186/1755-8794-5-28)
Supplement: Additional file 1 — Figure S1. Gene Ontology analysis on the differentially expressed genes. The top 13 highly significant classification/functions of genes are shown in the figure. [file 1755-8794-5-28-S1.ppt]

## Slide 1
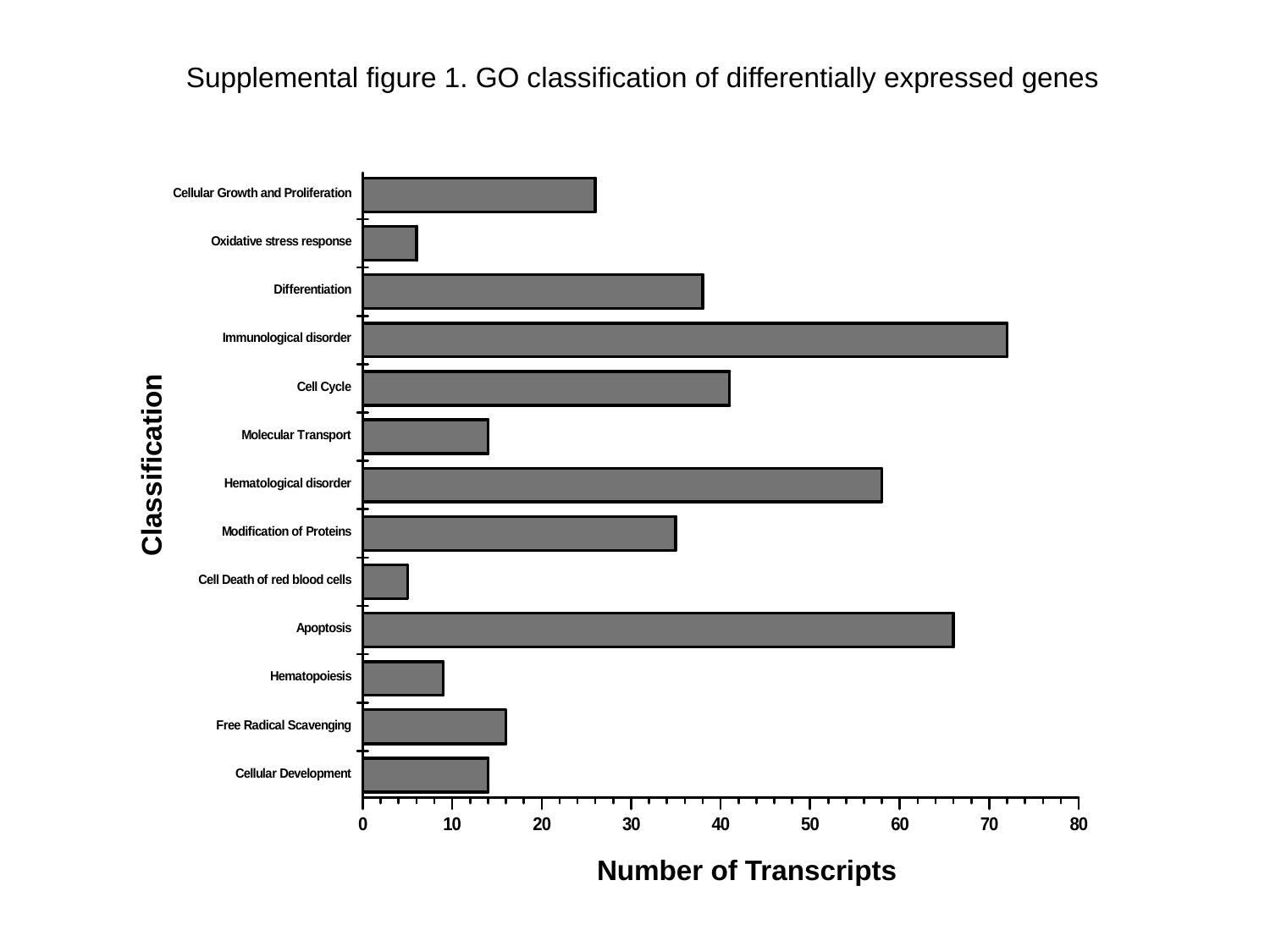

Supplemental figure 1. GO classification of differentially expressed genes
Classification
Number of Transcripts

## Slide 2
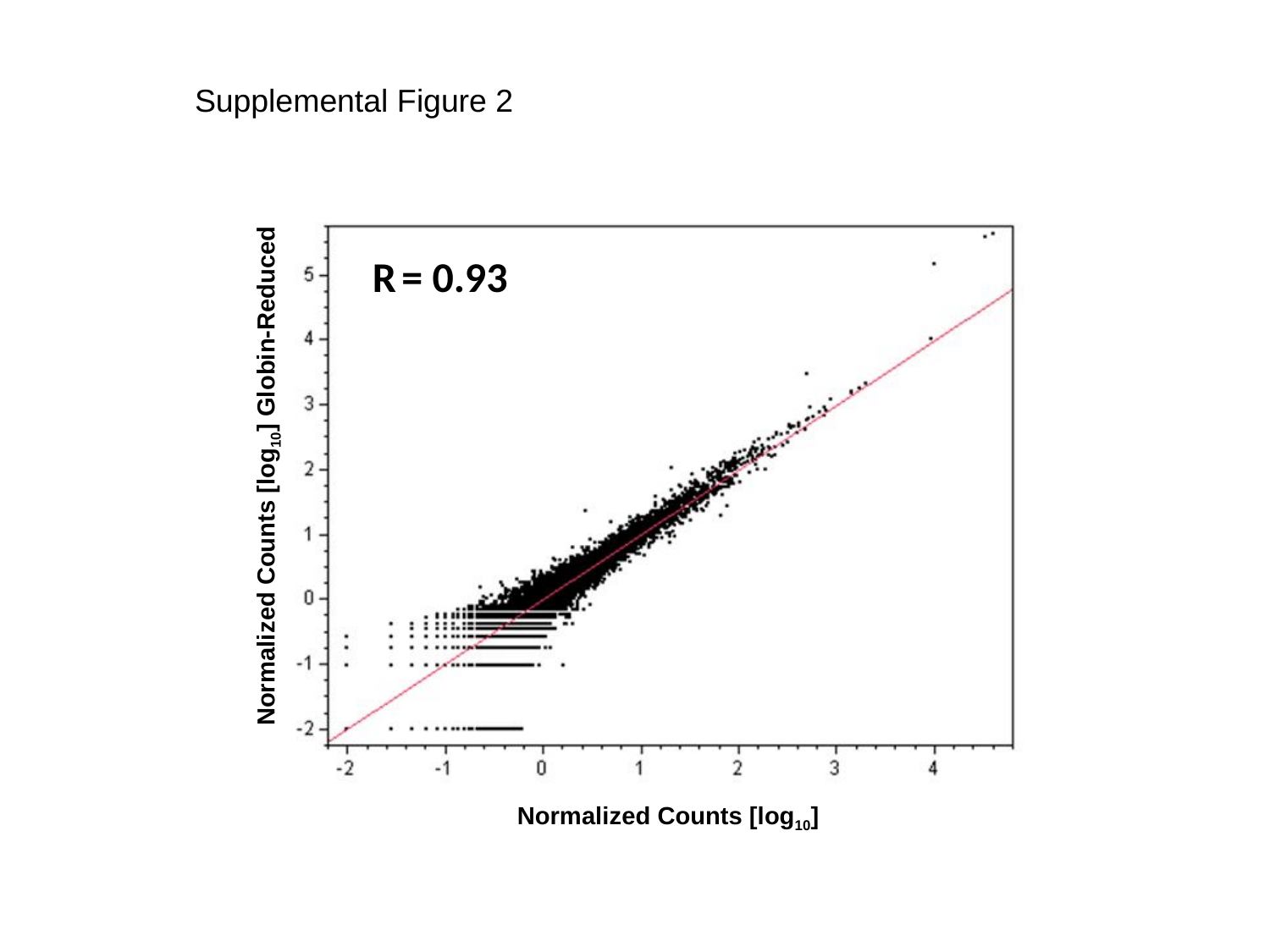

Supplemental Figure 2
R = 0.93
Normalized Counts [log10] Globin-Reduced
Normalized Counts [log10]
