# Supplementary figures and images for: A systematic comparison and evaluation of high density exon arrays and RNA-seq technology used to unravel the peripheral blood transcriptome of sickle cell disease
Source: BMC Med Genomics. 2012 Jun 29;5:28. doi: 10.1186/1755-8794-5-28 (PMC3428653; doi:10.1186/1755-8794-5-28)

## Slide 1
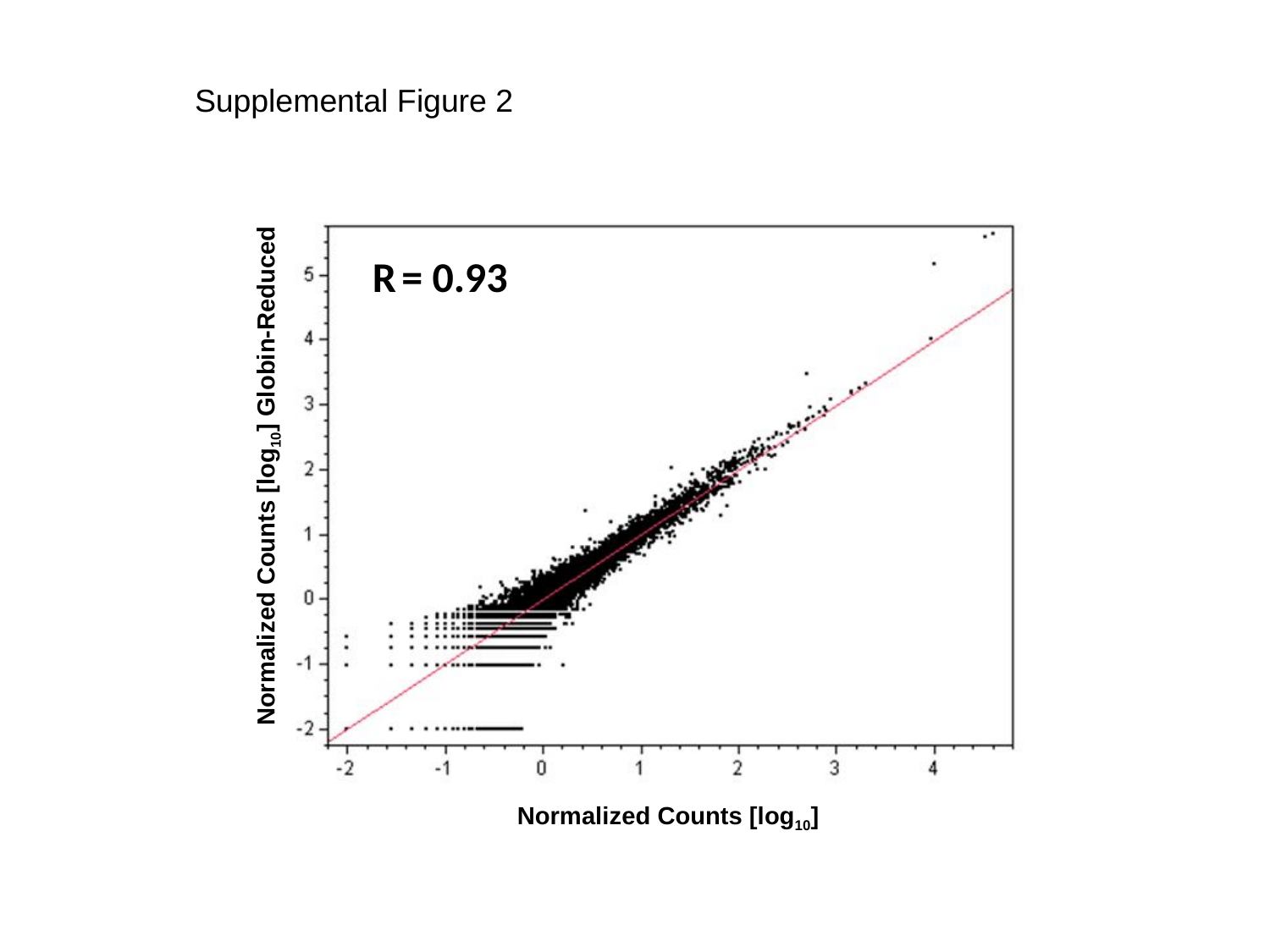

Supplemental Figure 2
R = 0.93
Normalized Counts [log10] Globin-Reduced
Normalized Counts [log10]

Supplement: Additional file 2 — Figure S2. Effect of Globin Reduction on RNA-seq expression. Y-axis: Read counts per transcript, normalized by median for globin reduced sample; X-axis: Read counts normalized by median for same sample using standard preparation. Correlation coefficient is 0.93. [file 1755-8794-5-28-S2.ppt]

## Slide 1
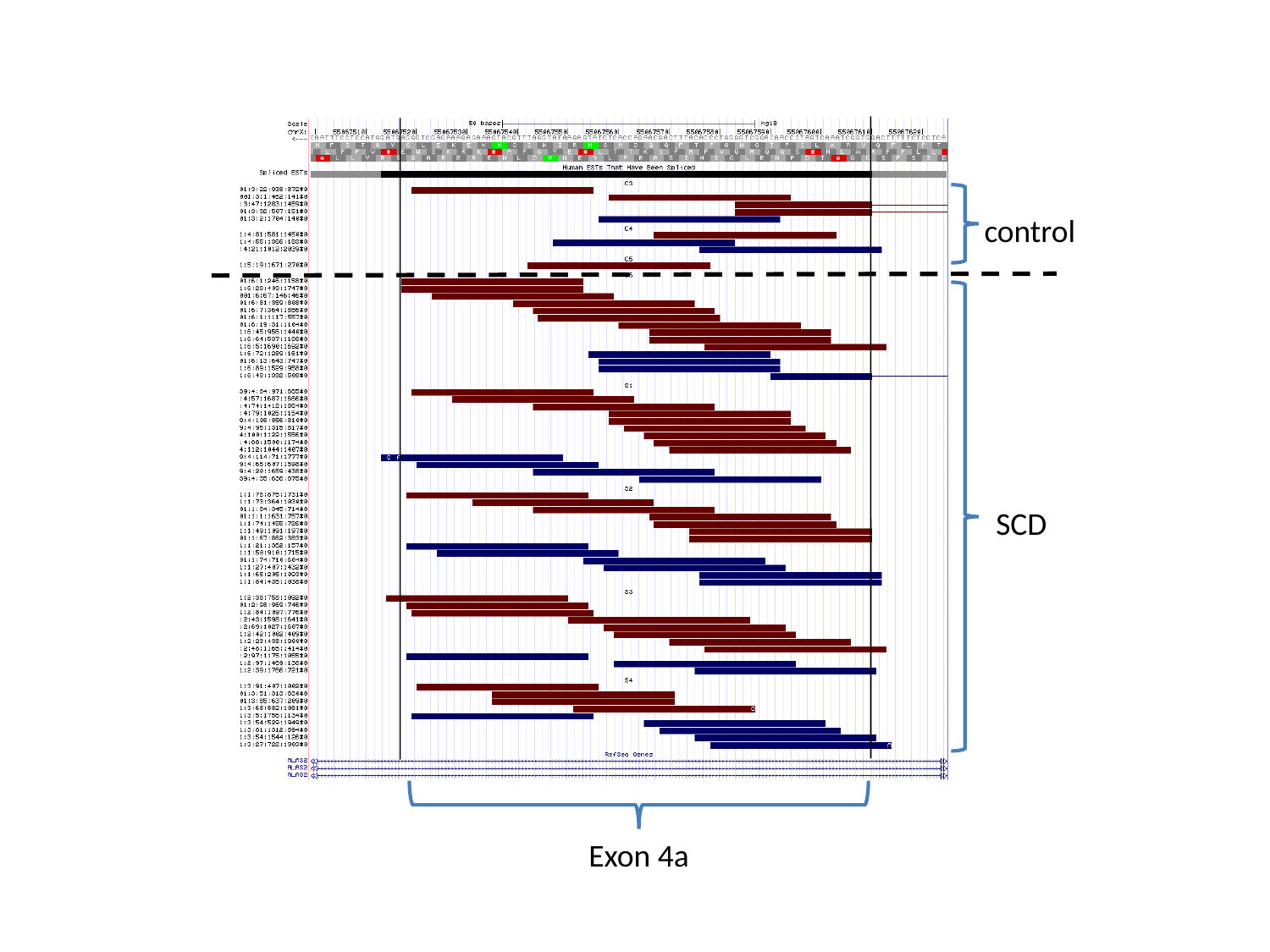

control
SCD
Exon 4a

Supplement: Additional file 4 — Figure S3. Putative Exon 4A . UCSC Genome Browser view of the BAM files for each sample showing genomic region chromosome X: 55067500–5506725. The first 3 aligned tracks show the control samples, the following 5 tracks show the sickle cell disease samples. Aligned reads are Red if to the negative strands and blue if to the positive strands. The total reads per sample is: C3: 15,715,705, C4: 15,131,360, C5: 15,730,372, S6: 16,570,843, S1: 13,481,528, S2: 16,707,788, S3: 14,650,161, S4: 18,580,778 and S5: 16,460,443. S6: 16570843, S1. [file 1755-8794-5-28-S4.ppt]
